# Upregulation of MUC5AC by VEGF in human primary bronchial epithelial cells: implications for asthma

**DOI:** 10.1186/s12931-019-1245-1

**Published:** 2019-12-12

**Authors:** Sung-Ho Kim, Qing-Mei Pei, Ping Jiang, Juan Liu, Rong-Fei Sun, Xue-Jiao Qian, Jiang-Bo Liu

**Affiliations:** 1 Department of Respiration, Tianjin First Central Hospital, Fukanglu-24, Nankaiqu, Tianjin, 300192 China; 2 Department of Radiology, Tianjin Hospital of Integrated Traditional Chinese and Western Medicine, Tianjin, China

**Keywords:** Asthma, Vascular endothelial growth factor, MUC5AC, Caveolin-1

## Abstract

**Background:**

Airway mucus hypersecretion is an important pathophysiological feature in asthma. Mucins are glycoproteins that are mainly responsible for the viscoelastic property of mucus, and MUC5AC is a major mucin glycoprotein that is overproduced in asthma. Vascular endothelial growth factor (VEGF) has been implicated in inflammatory and airway blood vessel remodeling in asthmatics. Therefore, we sought to investigate the effect of VEGF on MUC5AC expression and study the underlying mechanisms.

**Methods:**

In order to elucidate the precise mechanism underlying the effect of VEGF on MUC5AC expression, we tested the effects of VEGF on RhoA activation and the association of caveolin-1 and VEGFR2 in Primary Bronchial Epithelial Cells.

**Results:**

VEGF up-regulated MUC5AC mRNA and protein levels in a dose- and time-dependent manner, and activated RhoA. Additionally, VEGF-induced MUC5AC expression and RhoA activation were enhanced by disrupting caveolae with cholesterol depletion and reversed by cholesterol repletion, and inhibited by a selective VEGF receptor 2 (VEGFR2) inhibitor SU1498. Furthermore, phospho-VEGFR2 expression was decreased via overexpression of caveolin-1. VEGF treatment reduced the association of caveolin-1 and VEGFR2.

**Conclusion:**

Collectively, our findings suggest that VEGF up-regulates MUC5AC expression and RhoA activation by interaction with VEGFR2, and this phenomenon was related with the association of caveolin-1 and VEGFR2. Further studies on these mechanisms are needed to facilitate the development of treatments for asthma.

## Background

Airway mucus hypersecretion is now recognized as an important pathophysiological feature in asthma [1]. Excessive accumulation of airway mucus leads to the formation of mucous plugs that increase airway resistance and reduce the effective airway diameter. Mucins are glycoproteins that are mainly responsible for the viscoelasticity of the mucus. MUC2, MUC4, MUC5AC, and MUC5B proteins are the primary *mucins* in human airways. Among them, MUC5AC is a major mucin glycoprotein and is overproduced in asthma [2, 3].

Caveolae are flask-shaped plasma membrane specializations characterized by their high hydrophobicity. A multitude of signal transduction molecules, including caveolin-1, tyrosine kinase, Raf, MEK1/2, and transient receptor potential canonical channels, accumulate in the caveolae [4]. Recent data indicated that cultured primary bronchial epithelial cells (PBECs) of asthmatics had lower caveolin-1 expression compared to that in the control cells [5]. In vitro studies revealed that IL-4 causes aggregation of caveolin-1-containing lipid rafts, resulting in increased MUC5AC synthesis in bronchial epithelial cells.

It is well known that vascular endothelial growth factor (VEGF) is a potent stimulator of angiogenesis in asthma. Studies have revealed that VEGF levels are increased in lung tissues and sputum of asthmatic patients and positively correlate with asthma disease severity. Furthermore, inhibition of VEGF can lead to a significant reduction in goblet cell hyperplasia and basement membrane thickness [6]. Mucin protein-MUC5AC has been implicated as one of the markers of goblet cell metaplasia in lung pathologies [7].

Therefore, in the present study, we aimed to investigate the regulatory effect of VEGF on MUC5AC expression and elucidate the underlying mechanisms.

## Methods

### Antibodies and reagents

Antibodies against MUC5AC, RhoA, phospho-VEGFR2 (Tyr1175), caveolin-1, and VEGFR2 were purchased from cell signaling technology (Danvers, MA). Antibody against β-actin was obtained from Santa Cruz Biotechnology (Santa Cruz, CA). The secondary antibodies were obtained from (Jackson Immunoresearch, West Grove, PA). HA-1077, filipin III, cholesterol, and VEGF were purchased from Sigma-Aldrich (St. Louis, MO). SU1498 and cyclodextrin were from CalBiochem (La Jolla, CA).

### Cell culture

PBECs were obtained from the American Type Culture Collection (Manassas, VA, USA). Cells were grown in RPMI-1640 with 10% fetal bovine serum (FBS) and maintained at 37 °C in a humidified atmosphere of 5% CO2 and 95% air. All inhibitors were dissolved in dimethyl sulfoxide (DMSO; final concentration of 0.1%, vol/vol) and added to the medium. Vehicle controls contained the same amount of DMSO.

### Real-time reverse transcriptase–PCR

Total RNA was isolated from PBECs using an Easy-BLUE Total RNA Extraction Kit (iNtRON Biotechnologies, Shanghai, China) after exposure to VEGF. Total RNA (2 μg) was reverse transcribed using the oligo (dT) primer and MMLV reverse transcriptase (Promega, Madison, WI) at 42 °C for 90 min. Real-time PCR was performed using an ABI Prism 7500 instrument according to the manufacturer’s instructions (Applied Biosystems, Foster City, CA). The following primer pairs were used: MUC5AC, forward 5′- TCTGCAGCGAATCCTACTCG − 3′ and reverse, 5′- GGTTCTCTTCAATACGGGGG − 3′, and GAPDH, forward 5′- GGCCAAAAGG GTCATCATC − 3′ and reverse, 5′-GTGATGGCATGGACTGTGG-3′. After an initial hot start for 10 min, amplification was performed for 40 cycles consisting of denaturation for 10 s at 94 °C, annealing for 30 s at 56 °C, and extension for 40 s at 72 °C. The amplification kinetics was recorded as sigmoid progress curves for which fluorescence was plotted against the number of amplification cycles. The threshold cycle number (CT) was used to define the initial amount of each template. The CT was the first cycle for which a detectable fluorescent signal was observed. The mRNA expression levels were determined and compared with the GAPDH standard.

### Western blot analysis

The cell extracts were separated by 10% sodium dodecyl sulphate-polyacrylamide gel electrophoresis (SDS-PAGE) and transferred onto a nitrocellulose membrane. The membranes were blocked in blocking solution [5% non-fat dried milk in phosphate buffered saline (PBS)] for 2 h at room temperature and then probed with anti-MUC5AC, anti-Rho A, anti-phospho-VEGFR2, anti-VEGFR2, anti-caveolin-1, and anti-β-actin for 1 h at room temperature. After washing three times in phosphatebuffered saline (PBS) containing 0.1% Tween-20 (PBS-T), the membranes were incubated with secondary antibodies for 1 h at room temperature. After washing an additional three times in PBS-T, the membranes were developed using an electrochemiluminescence (ECL) solution (Pierce, Rockford, IL, USA) and exposed to Kodak X-ray film.

### Transfection of small interfering RNA (siRNA)

RhoA was transfected into PBECs according to a siRNA transfection protocol provided by Ambion (Austin, TX, USA). Briefly, after culturing PBECs in antibiotic-free RPMI-1640 at 37 C in a humidified atmosphere of 5% CO2 for 24 h, the siRNA duplex solution, which was diluted in siRNA transfection medium (Santa Cruz Biotechnology), was added to the PBECs. After transfection for 24 h, the medium was replaced with normal RPMI-1640, and PBECs were treated with VEGF. Scrambled siRNA, purchased from Santa Cruz Biotechnology, was transfected to PBECs as a negative standard.

### RhoA activity assays

The activity of RhoA was assessed in PBECs by a pull-down assay for GTP-bound RhoA as previously described [8]. GTP-bound RhoA was precipitated from cell lysates with Rhotekin RBD (Upstate Biotechnology, Lake Placid, NY). After whsh the beads, and then the immunoprecipitate was resolved on 15% SDS-PAGE. Using an anti-RhoA antibody to detect active RhoA and total RhoA .

### Establishment of caveolin-1 overexpressing cell lines

To generate caveolin-1 overexpressing vectors, the caveolin-1-coding sequences were obtained by reverse transcription PCR and cloned into pMXs-based retroviral plasmid (Addgene). PBECs were infected as described [9], to establish caveolin-1 overexpressing PBECs (PBECs - Caveolin-1), and PBECs infected with retrovirus containing blank pMXs vector (PBECs - vector) were used as the control group.

### Immunoprecipitation

Immunoprecipitation and immunoblotting with anti-caveolin-1 and anti-VEGFR2 antibodies were performed as previously described [10]. After clarification, equal amounts of lysate were incubated overnight with 2 g of primary antibody rotating at 4 °C. With the exception of the agarose-conjugated anti-VEGFR-2 and anti-caveolin antibodies, the immune complexes were collected by incubating the mixtures with 25 μl (50% suspension) of Protein A (rabbit primary antibody) or Protein G (mouse primary antibody) Sepharose beads. Immunoprecipitates were extensively washed, resuspended in 2 sample buffer, boiled, and resolved by SDS-PAGE.

### Statistical analysis

All results are expressed as the mean ± SEM. The statistical evaluation of the results was performed by an independent t-test and an ANOVA with a Tukey post-hoc test.

The results were significant with a value of *p* < 0.05.

## Results

### VEGF up-regulates MUC5AC expression in PBECs

**Fig. 1:**
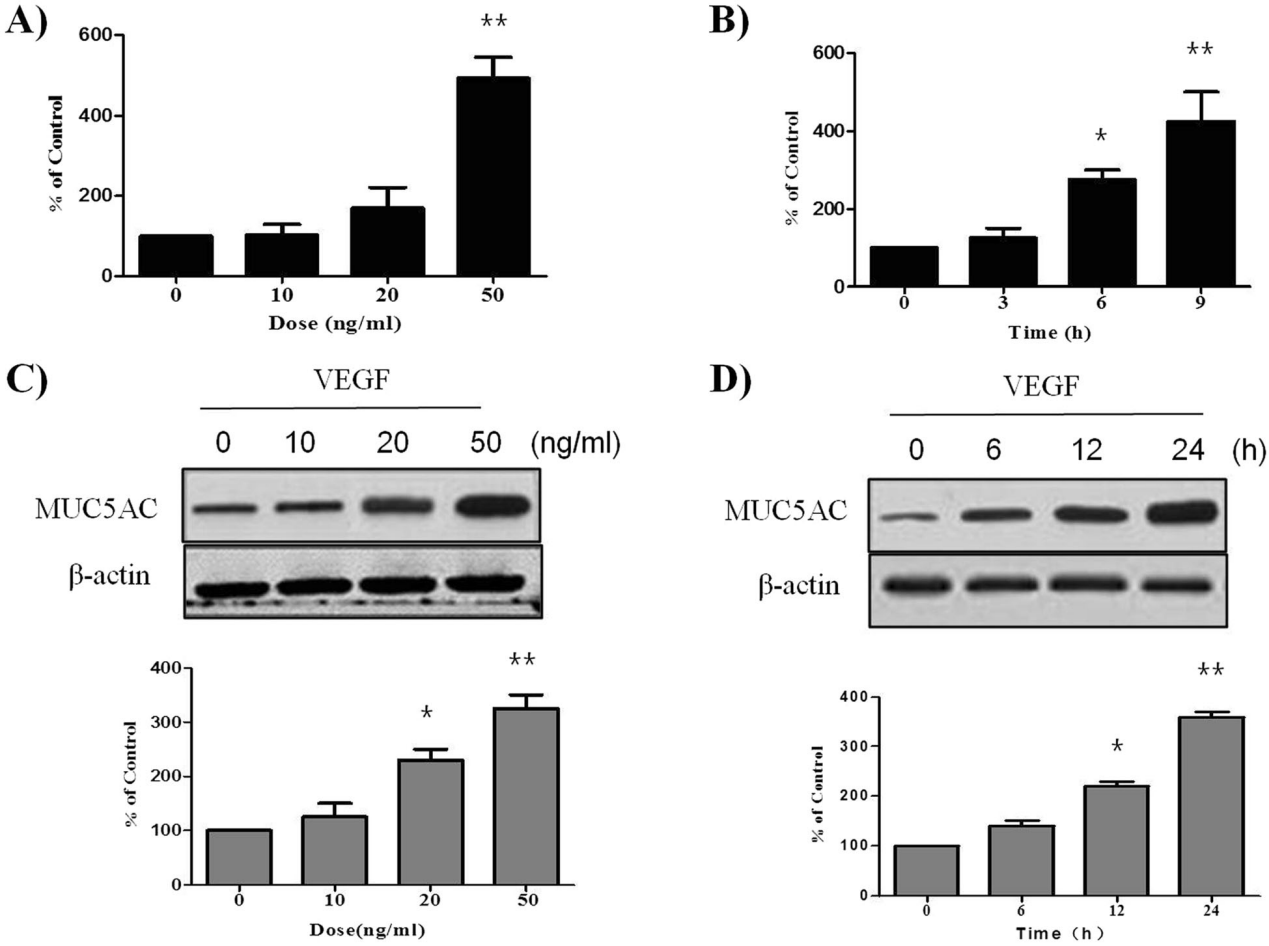
VEGF promotes MUC5AC expression at both mRNA and protein level. PBECs were incubated with indicated doses of VEGF for 9 h, and then real-time PCR performed. The values are normalized relative to the GAPDH standard (A). PBECs were incubated at indicated times of VEGF (50 ng/ml), and then real-time PCR performed (B). PBECs were incubated with indicated doses of VEGF for 24 h (C). PBECs were incubated at indicated times of VEGF (50 ng/ml), and then western blotting analysis for MUC5AC was performed. β-actin was used as a loading control (D). All data are representative of three independent experiments. The blots were quantified by densitometry. Values represent the means ± SEM. ^*^P < 0.05, ^**^P < 0.005 vs. control; *n* = 3

To evaluate the effect of VEGF on MUC5AC expression, we performed real-time PCR in PBECs. When PBECs were treated with various doses of VEGF for different times, MUC5AC mRNA expression was up-regulated in a dose- and time-dependent manner (Fig. 1a,b), suggesting that VEGF up-regulates MUC5AC mRNA expression in PBECs. In addition, we performed western blot analysis to evaluate whether VEGF up-regulates MUC5AC protein expression in PBECs. Interestingly, VEGF up-regulated MUC5AC protein expression in PBECs in a dose- and time-dependent manner (Fig. 1c,d). We did not find any VEGF-induced cytotoxicity under our experimental conditions (data not shown).

### **VEGF-induced** MUC5AC **upregulation depends on RhoA/rho kinase activation**

**Fig. 2:**
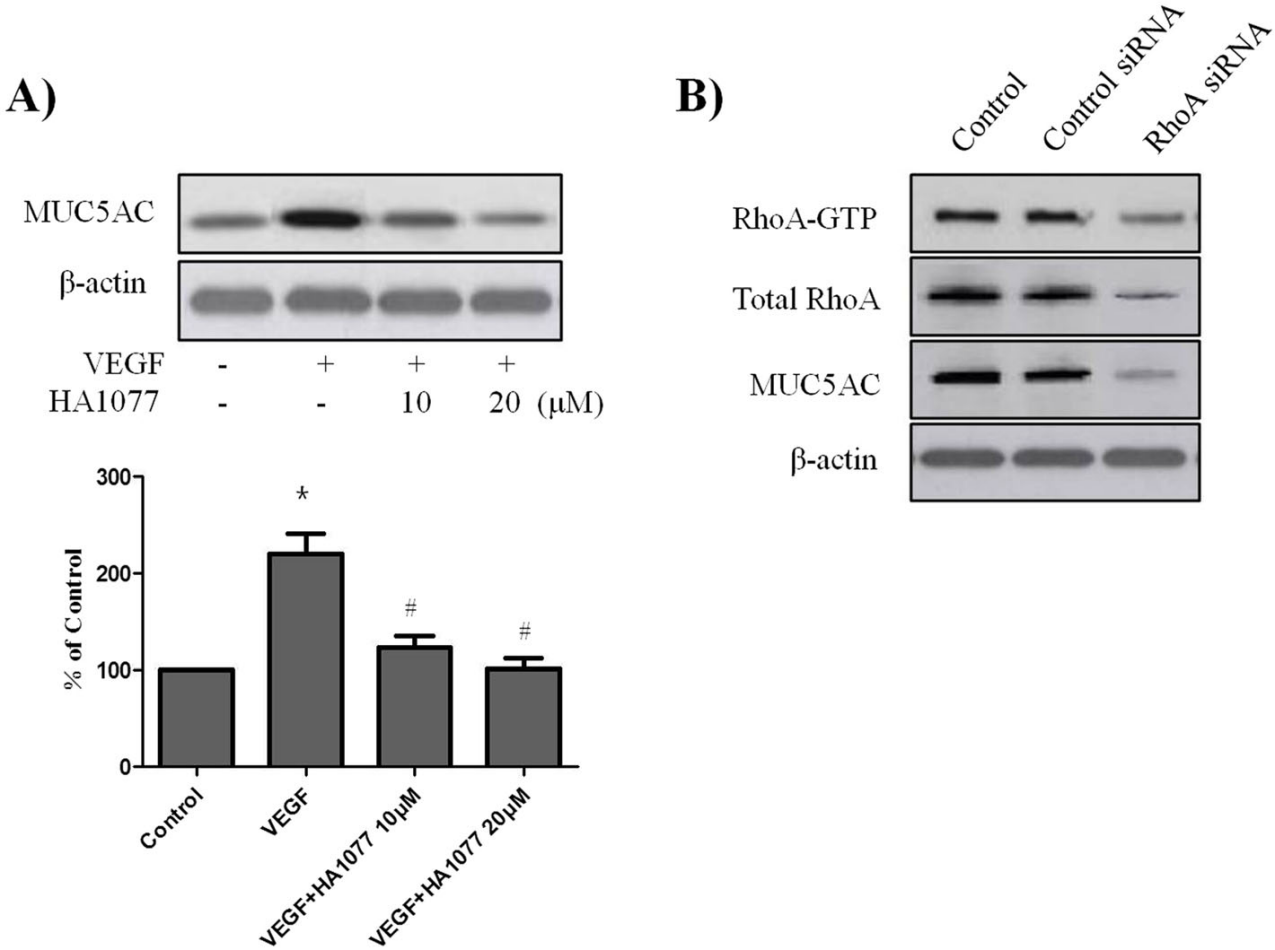
VEGF-induced MUC5AC upregulation depends on RhoA/Rho kinase activation. PBECs were incubated with indicated doses of Rho kinase inhibitor HA-1077 for 2 h before treatment with VEGF (50 ng/ml) for 24 h, and then western blotting analysis for MUC5AC was performed. β-actin was used as a loading control (A). PBECs were transfected with Control siRNA or RhoA siRNA, and then western blotting analysis for RhoA-GTP, Total RhoA, and MUC5AC was performed. β-actin was used as a loading control (B). All data are representative of three independent experiments. The blots were quantified by densitometry. Values represent the means ± SEM. ^*^P < 0.05 vs. control; ^#^ *P* < 0.05 vs. VEGF alone; *n* = 3

Simvastatin has been shown to attenuate airway mucus hypersecretion induced by LPS, and inhibitory effect of simvastatin may be through, at least in part, the inactivation of RhoA and p38 signaling pathway [11]. However, the involvement of RhoA activation in VEGF-induced MUC5AC upregulation has not yet been assessed. To evaluate whether RhoA/Rho kinase mediates VEGF-induced MUC5AC upregulation, PBECs were exposed to VEGF (50 ng/ml) in the presence of the Rho kinase inhibitor HA-1077, and MUC5AC protein levels were assessed by Western blot. The data in Fig. 2a shows that VEGF stimulation failed to increase MUC5AC protein levels in HA-1077-pretreated cells. Next, to confirm the involvement of RhoA activation in VEGF-induced MUC5AC upregulation, we constructed a RhoA siRNA transfection reagent. As shown in Fig. 2b, we confirmed RhoA gene silencing at the total protein level and activated form. When PBECs were transfected with RhoA siRNA or control siRNA for 48 h, MUC5AC expression was decreased in RhoA siRNA transfected cells compared to that in the negative control siRNA transfected cells. These data indicate a critical role for RhoA/Rho kinase activation in VEGF-induced MUC5AC upregulation.

### Caveolae are critical for VEGF-induced RhoA activation and MUC5AC upregulation

**Fig. 3:**
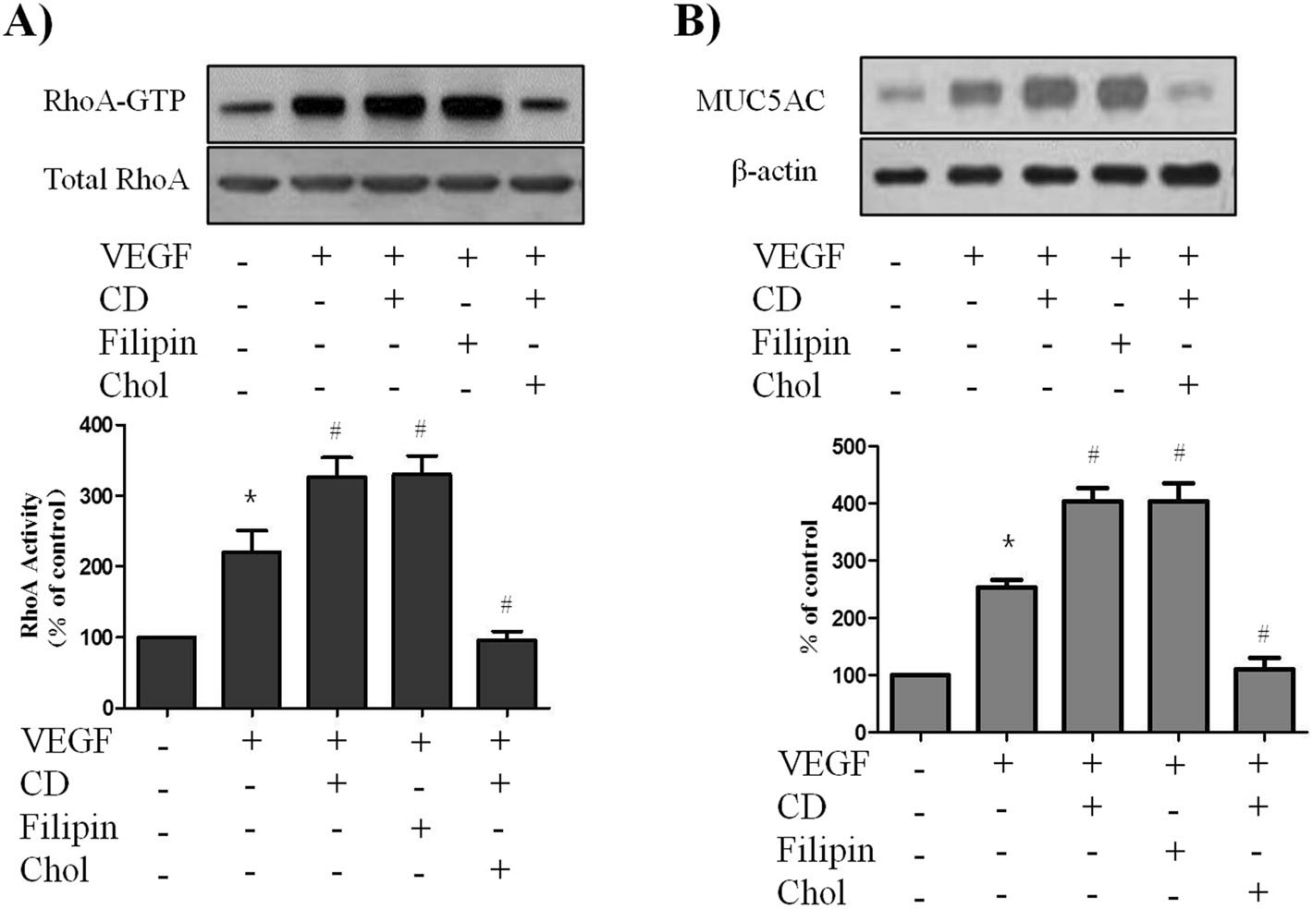
Caveolar disruption enhanced the VEGF-induced RhoA activation and MUC5AC up-regulation. PBECs were treated with VEGF (50 ng/ml) in the presence or absence of pretreatment with the caveolar-disrupting agent cyclodextrin (CD; 5 mM, 30 min) or filipin (2.5 g/ml, 30 min). Reversal of drug effects was sought with simultaneous cholesterol repletion (Chol; 15 g/ml) given at the time of cyclodextrin administration. RhoA activity was assessed by pull-down assay of GTP-bound RhoA (24 kDa) as described in Method (A). MUC5AC protein levels were assessed by Western blot (B). All data are representative of three independent experiments. The blots were quantified by densitometry. Values represent the means ± SEM. ^*^P < 0.05 vs. control; ^#^ *P* < 0.05 vs. VEGF alone; *n* = 3

It has been reported that PKC-mediated contraction and Rho activation are increased in smooth muscle following genetic ablation of caveolin-1 [12], we thus examined the effects of caveolar disruption on VEGF-induced RhoA activation and MUC5AC upregulation. We used the membrane-impermeable cholesterol-binding agent cyclodextrin, which depletes cell surface cholesterol, and the membrane-permeable agent filipin III to perturb the formation of caveolae. We observed that both cyclodextrin and filipin III enhanced VEGF-induced RhoA activation (Fig. 3a). We further tested whether the effect of cyclodextrin was reversible by coincubation with excess cholesterol. As shown in Fig. 3a, cholesterol reverses the effects of cyclodextrin on RhoA activation. Similar results were observed for MUC5AC protein upregulation (Fig. 3b). These data suggest that VEGF-induced RhoA activation and MUC5AC upregulation depend on the structural integrity of caveolae in PBECs.

### VEGF-induced **RhoA activation** and MUC5AC **upregulation** is dependent on VEGFR2 (KDR/Flk1)

**Fig. 4:**
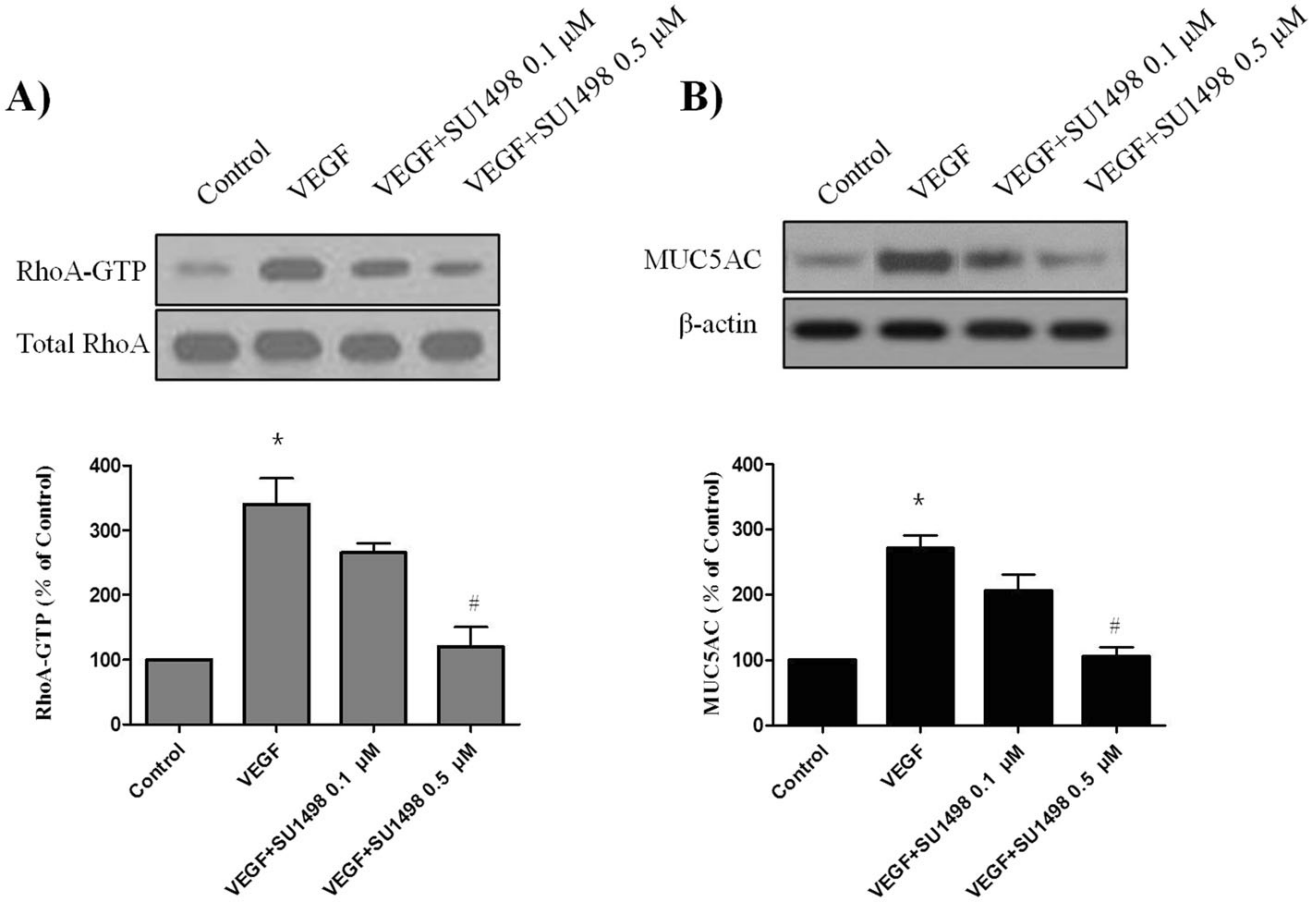
Effect of SU1498 on VEGF-induced RhoA activation and MUC5AC expression in PBECs. PBECs were incubated with indicated doses of SU1498 for 2 h before treatment with VEGF (50 ng/ml) for 24 h, and then western blotting analysis for RhoA activation was performed. Total RhoA was used as a loading control (A). PBECs were incubated with indicated doses of SU1498 for 2 h before treatment with VEGF (50 ng/ml) for 24 h, western blotting analysis for MUC5AC activation was performed. β-actin was used as a loading control (B). All data are representative of three independent experiments. The blots were quantified by densitometry. Values represent the means ± SEM. ^*^P < 0.05 vs. control; ^#^ *P* < 0.05 vs. VEGF alone; *n* = 3

As VEGF-induced RhoA activation depends on the activity of VEGFR2 in mesangial cells, we sought to determine whether blocking the VEGF-VEGFR2 interaction will prevent VEGF-induced RhoA activation and MUC5AC protein upregulation in PBECs. We used SU1498, an inhibitor of the tyrosine kinase activity of VEGFR2 that blocks the interaction of VEGF with VEGFR2, but not with VEGFR1 (FLK1). As shown in Fig. 4a, VEGF-induced RhoA activation was inhibited by SU1498 in a dose-dependent manner. In addition, SU1498 also blocked VEGF-induced MUC5AC protein upregulation in PBECs (Fig. 4b). These data suggest that VEGF-induced RhoA activation and MUC5AC upregulation depend on the VEGF/VEGFR2 interaction in PBECs.

### Caveolin-1 is a negative regulator of VEGFR-2 activity

**Fig. 5:**
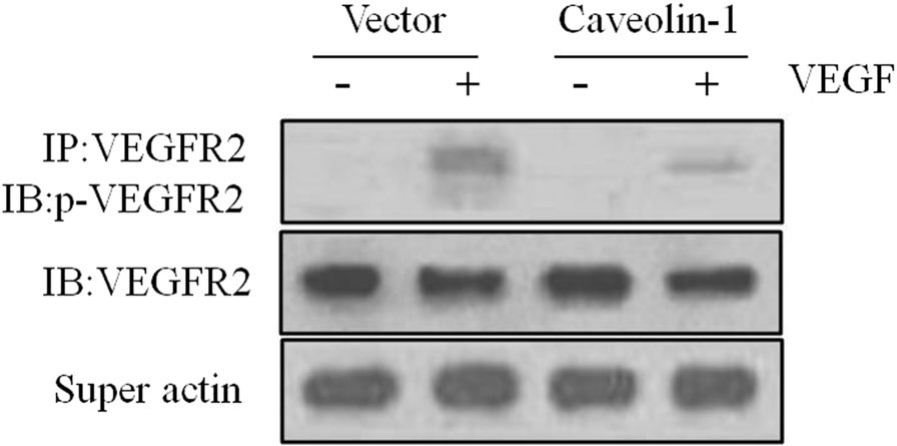
Transfection of caveolin-1 in PBECs inhibits VEGFR2 phosphorylation. PBECs were transiently transfected with caveolin-1 cDNAs. Forty-eight hours posttransfection, cells were serum-starved and lysed. VEGFR-2 was immune- precipitated from cell extracts and phosphorylation was monitored by Western blotting with an anti-phospho-VEGFR2 antibody. Actin in the supernatant was probed to ensure equal immunoprecipitation across conditions Data was representative of three independent experiments

We further investigated the regulatory function of caveolae on VEGF-dependent signaling by examining the effect of caveolin-1 on VEGFR-2 activity. Interestingly, overexpression of caveolin-1 resulted in a marked inhibition of VEGFR-2 activity compared to that in the vector control (Fig. 5). These data suggest that VEGF-induced RhoA activation and MUC5AC upregulation depend on the caveolin-1/VEGFR2 interaction in PBECs.

### VEGFR2 association with caveolin-1 is markedly reduced with VEGF pretreatment

**Fig. 6:**
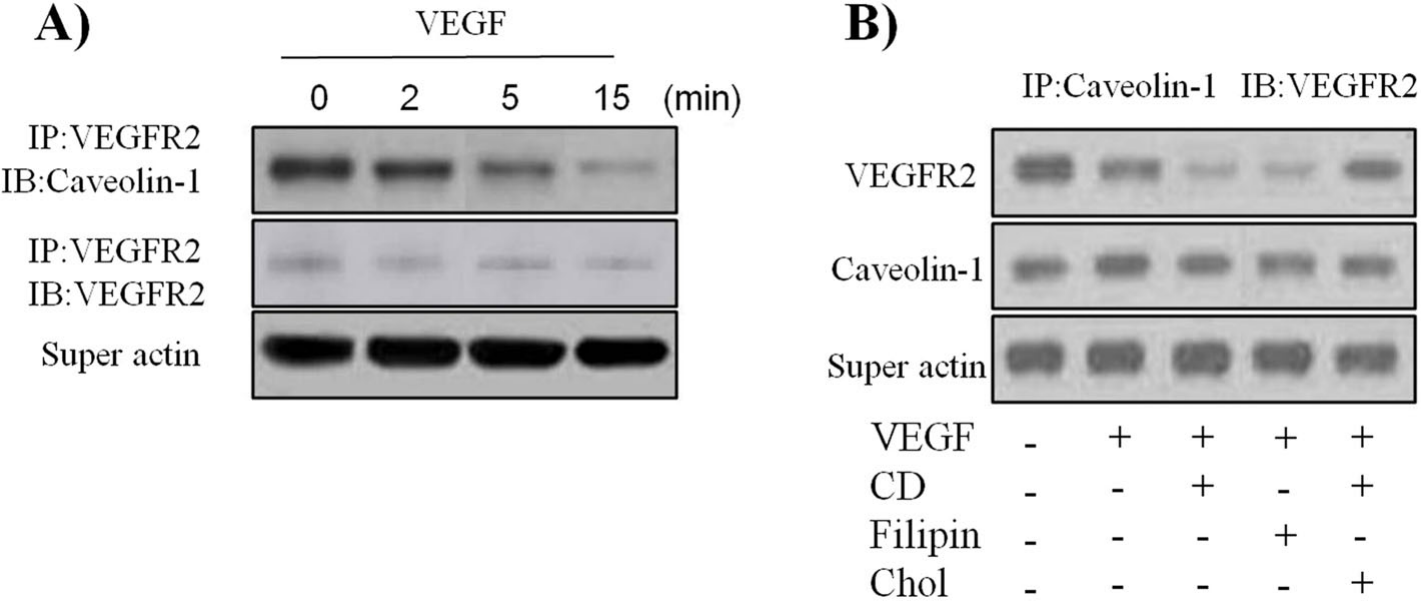
VEGFR2 association with caveolin-1 is markedly decreased by treatment with VEGF, cyclodextrin, or filipin and is partially rescued by treatment with cholesterol repletion. VEGFR2 was immunopricipitated from PBECs lysates, and its association with caveolin-1 was assessed by Western blot. Actin in the supernatant was probed to ensure equal immunoprecipitation across conditions (A). PBECs were treated with VEGF (50 ng/ml) in the presence or absence of pretreatment with the caveolar-disrupting agent cyclodextrin (CD; 5 mM, 30 min) or filipin (2.5 g/ml, 30 min). Reversal of drug effects was sought with simultaneous cholesterol repletion (Chol; 15 g/ml) given at the time of cyclodextrin administration. Caveolin-1was immunopricipitated from PBECs lysates, and its association with VEGFR2 was assessed by Western blot. Actin in the supernatant was probed to ensure equal immunoprecipitation across conditions (B). All data are representative of three independent experiments

It has been reported that caveolin-1 colocalizes with VEGFR2 on the plasma membrane caveolae in endothelial cells [10, 13], although this has not yet been demonstrated in PBECs. To evaluate whether or not an association has been made between the caveolin-1 and VEGFR2, we performed an immunoprecipitation experiment. As shown in Fig. 6a, in unstimulated PBECs, caveolin-1 protein was detected in anti-VEGFR2 immunoprecipitates from protein extracts. Interestingly, the stimulation of PBECs with VEGF induced a rapid, time-dependent dissociation of caveolin-1 from VEGFR2, this dissociation being nearly complete 15 min after the addition of VEGF. We also investigated the effects of cholesterol depletion on the association of VEGFR2 and caveolin-1. The VEGFR2/caveolin-1 association decreased by VEGF was further reduced with pretreatment with cyclodextrin and filipin (Fig. 6b). These results indicate that under resting conditions, caveolin-1 may act as a negative regulator of VEGFR2 activity and that stimulation of the receptor by VEGF may promote activation of this signaling pathway by inducing the dissociation of the receptor from the inhibitory action of caveolin-1. We also observed that the addition of cholesterol reversed the effects of cyclodextrin on VEGFR2/caveolin-1 association (Fig. 6b). These data suggest that the impaired caveolin-1 expression in PBECs upregulates VEGF signaling, and this may be principally mediated by VEGFR2/caveolin-1 association and intact caveolae as upstream regulators.

## Discussion

Understanding the regulatory mechanisms of airway mucus hypersecretion is of potential clinical value and can provide further insights into new treatment strategies of airway diseases such as asthma. MUC5AC is a major mucin glycoprotein that is overproduced in asthma [3]. MUC5AC is upregulated in response to inflammation of the lungs caused by disorders such as bronchial asthma. MUC5AC expression and secretion are induced by pro-inflammatory cytokines such as IL-4, IL-13, and transforming growth factor (TGF)-a, and by external agents, such as viruses and cigarette smoke. However, no study has yet reported the effect of VEGF on MUC5AC expression and secretion.

VEGF is a well-known, potent stimulator of angiogenesis in asthma. Elevated VEGF levels have been observed in lung tissues and sputum of asthmatic patients and positively correlate with asthma disease severity. Furthermore, inhibition of VEGF can lead to a significant reduction in basement membrane thickness and goblet cell hyperplasia [6]. Recent studies have found increased levels of MUC5AC in total protein extracts from lung tissues of asthmatic mice. This increase was reduced substantially by the administration of SU5614 [7]. Therefore, we investigated the effects of VEGF and relevant signal transduction pathways on MUC5AC expression in human PBECs. Further research is needed to perform *animal experiment to support* a direct effect of VEGF on the MUC5AC expression.

Caveolae are flask-shaped plasma membrane invaginations that are abundant in cholesterol and sphingolipids. Caveolin-1 is the major structural protein of the caveolae, and plays a key role in the formation and mobility of these structures. Studies have found that endobronchial biopsies show a remarkable decrease in caveolin-1 levels in the lungs of asthmatic patients compared to those in the controls. This loss was most evident in bronchial epithelial cells. Furthermore, studies involving cultured PBECs of asthmatics showed lower caveolin-1 expression compared to that in the control cells [5]. It has been reported that Rho-kinase is associated with airway mucus secretion in patients with asthma. In addition, Rho-A/Rho kinase inhibitor, fasudil, reduced mucous secretion and MUC5AC expression in OVA-challenged mice [14]. Rho activation is increased in smooth muscle following genetic ablation of caveolin-1 [12]. Recent studies have shown that caveolae are clearly required for VEGF-induced activation of RhoA [15]. Consistent with this report, we also found that VEGF-induced RhoA activation and MUC5AC upregulation depend on the structural integrity of caveolae.

VEGF signaling activation involves its binding to VEGFR2 and consequent internalization into the cytoplasm. The signaling of VEGF starts after VEGFR2 internalization and the fate of activated receptors depends on its transport to late endosome for degradation, or alternatively for recycling. It has been reported that both VEGFR2 and caveolin-1 are localized on caveolae in several cell lines [10, 13, 16]. Several studies, carried out on different receptor tyrosine kinases, suggest that the transport to late endosome should be mediated by caveolin-1 and a decrease of caveolin-1 may result in enhanced growth factor signaling [17, 18]. The localization of both receptors and signaling partners to caveolae has been considered a pivotal mechanism to control the levels of both receptors and signaling proteins, their availability, and activation [18]. Recent studies observed that caveolin-1 is associated with the inactive form of VEGFR2 and undergoes rapid dissociation from the receptor upon stimulation with VEGF. This association of caveolin-1 is inhibitory to VEGFR2 activity [10]. In the present study, we found that overexpression of caveolin-1 led to a marked inhibition of VEGFR-2 activity and that the VEGFR2/caveolin-1 association decreased by VEGF treatment. Regarding the VEGFR2 internalization, two different pathways have been postulated: the caveolae-mediated endocytosis and the clathrin-mediated internalization [19]. The central question of whether or not VEGFR2 uses caveolar pathway for internalization could not be clearly answered by our present study; further research is needed to elucidate the signaling mechanisms.

## Conclusions

To our knowledge, we provide the first evidence on the role of caveolin-1/VEGFR2 on VEGF-induced RhoA activation and MUC5AC upregulation in bronchial epithelial cells, suggesting an effective therapeutic target in inflammatory diseases such as asthma.

## Data Availability

Source data and material will be made available upon reasonable request.

## Abbreviations

PBECs: Primary bronchial epithelial cells
VEGF: Vascular endothelial growth factor
VEGFR: Vascular endothelial growth factor receptor
